# Is Radical Doubt Morally Wrong?

**DOI:** 10.1007/s10670-024-00799-3

**Published:** 2024-05-01

**Authors:** Chris Ranalli

**Affiliations:** Department of Philosophy, VU Amsterdam, De Boelelaan 1105, Amsterdam, The Netherlands

## Abstract

Is radical skepticism ethically problematic? This paper argues that it is. Radical skepticism’s strong regulation of our doxastic economy results in us having to forego doxastic commitments that we owe to others. Whatever skepticism’s epistemic defects, it is ethically defective. In turn, I defend Moralism, the view that the kind of extreme doubt characteristic of radical skepticism is a serious moral and eudaimonic weakness of radical skeptical epistemology. Whether this means that skepticism is false or incorrect, however, is a further claim that Moralists may or may not accept. I distinguish between an encroachment and abrogation version of the view, and show how each one bears on radical skepticism. In either case, Moralism makes our beliefs less vulnerable to radical revision. The paper concludes with some exploratory reflections on whether the argument can be extended to show that radical skepticism is politically problematic, even risking injustice.

## Introduction

Much recent work on skepticism has turned to its practical and applied dimensions (Carter & McKenna, 2020; Gardiner, 2021; Hannon, 2019; McCormick, 2021; Rinard, 2021; Worsnip, 2021). Here, I want us to explore some of skepticism’s ethical dimensions. Imagine someone who, convinced that they ought not believe that there are any external things, other minds, past events, and so forth, no longer has any such beliefs. Could such a person, insofar as they otherwise lead an average person’s life—living the same way if they had such beliefs—nevertheless lead an ethical life? This paper explores that question. I argue that radical skeptics face a dilemma. Either they can’t ethically be skeptics, or else they eudaimonically weaken their lives as skeptics. Put most anemically, there’s something ethically problematic with skepticism that goes beyond any distinctively *epistemic* mistake.^1^

But does finding out that skepticism is ethically problematic have any bearing on its correctness? Not necessarily. Perhaps being a skeptic is wrongful or eudaimonically bad and maybe it’s true that no one is justified in believing anything about the external world. I later explore whether the dilemma I set out for skepticism and would-be extreme doubters (something I’ll speak to in a moment) means that we ought to reject skepticism. As we’ll see, there are ways of motivating a stronger and weaker ethical response to skepticism.

But why approach skepticism from this angle anyway? While most philosophers think that skepticism is worrying because it threatens to deprive us of epistemic goods—knowledge, justification, warrant—that’s not the only reason why it’s worrying. Many think it would be “just plain depressing” if it were true (Hannon, 2019, 143; cf. Rinard, 2021). Our key thought develops this idea. Skepticism is depressing because of what it would mean for how we live our lives.

As a preview of the argument, skeptics tell us that we justifiably believe that *p* only if we can eliminate every possibility we know to be incompatible with it. Unfortunately, this principle captures far too many personal beliefs within its net. You justifiably believe that your children love you only if you justifiably believe that they exist, for example. But, say skeptics, you can’t eliminate the possibility that you’re merely being tricked by an evil demon.^2^ So, you don’t justifiably believe that your children exist, and so you don’t justifiably believe that they love you either. And yet if you don’t justifiably believe that your children love you—absent other weightier considerations—you ought to lose your belief that your children love you. After all, if the belief is unjustified, doesn’t that tell against it? (More on this in §2–3). And insofar as you continue to relate to them, this suggests that you thereby ought to do something that would be undeservedly hurtful—to stop believing that they love you—or else forego these intimate relationships, which seems like a serious deprivation. Hence, radical skepticism is either morally wrongful or quite seriously bad for you. This dilemma pressures the skeptic to reconsider their position or else to strengthen it in problematic ways, as we’ll see. If their practice is morally risky or even wrongful, that raises the stakes of their position; it’s not like the commitment to abstract objects, or the institutional theory of art. And if it imposes serious limitations on their own lives, this too can lead them to amend their position, or rethink its value.^3^,^4^

We’ll proceed as follows. §2 states our preliminaries and centers on analogies between radical skepticism and high-standards that may lead some to doubt in cases of rape accusations, trolling, and hyper-individualism. §3 explains how skeptical principles erode our interpersonal beliefs. §4 presents the main argument. §5 defends its premises. §6 clarifies our commitments, the  differences between my view and similar views (e.g., Hirsch, 2018; Rinard, 2021) and answers questions about the extent to which the view adequately addresses radical skepticism. The conclusion (§7) briefly explores the extent to which the argument generalizes for social-political beliefs to highlight why radical skeptics might be non-neutral in a politically problematic way.

## Preliminaries

Call ‘Moralism’ the view that radical skepticism is in some way ethically wrong or bad. By ‘ethical’ I mean to encompass morality and features of the good life, like flourishing and well-being. This paper defends Moralism. As developed here, however, the idea is not that radical skepticism is categorically morally wrong—that any world where skepticism holds must also be a world where some ethical wrongness is instantiated—but that skepticism is conditionally morally wrong; conditional on undertaking certain ordinary actions, and otherwise *bad*: a kind of personal harm or deprivation.^5^

Moralism is also a kind of pragmatic response to skepticism. Pragmatic responses to skepticism say that there are pragmatic reasons for opposing skepticism (McCormick, 2021; Rinard, 2021). For example, that it is practically rational to believe that there is an external world or to believe propositions which commit us to that. A *pragmatic reason* is a reason which serves one’s practical interests (Meylan, 2021, 3).^6^ Pragmatic responses to skepticism divide into two. There are those that say it is practically impossible to *be* a skeptic and those that say we have pragmatic reasons to *believe *what we do. Moralism, as developed here, is a species of the former kind. I say: maybe you can be a skeptic, just not ethically or in a eudaimonically good way. The Moralism defended here can best be understood against the following backdrop:

*A* stands in practical relations to her family, friends, and other loved ones, believing, for example, that she loves them and believing that they, for example, love her. A counterpart to *A*, A* stands in the same practical relations to her family, friends, and other loved ones, and is like *A* in every other way, except that she doesn’t have any beliefs or positive credence of the relevant kind that befit her practical relations.

Moralism here says that there is an important ethical difference between *A* and A*. The connection to skepticism is that skepticism implies that you should be like A*, or else give up the relevant practical relations. But being like A* seems immoral; and systematically giving up those kinds of practical relations doesn’t seem so good either, or so I will argue.

Who is Moralism for? Moralism can sway those who are non-skeptical albeit attracted to the skeptic’s arguments.^7^ Even if skeptical epistemology is correct, this doesn’t mean that we should thereby become radical skeptics. Its ethical costs make this more plausible in a way that mere inconvenience or other practical considerations would not (§6). Moralism also reveals for skeptics why their position is ethically costly, something overlooked in the contemporary debate.^8^ While this might not convince skeptics to change their position—which is a questionable desideratum anyway—it presses them to take ownership for it, either strengthening their position or else embracing its costs, deepening its implausibility, as we will see.^9^

Nozick characterized skepticism as “extreme” (Nozick, 1981, 197). Focusing on the extreme doubter, a practical proxy for the radical skeptic, can help us get the feel of Moralism. The extreme doubter incessantly doubts what other people tell him. Consider Henry, who values a hyper-individualistic principle of autonomy, whereby you rely only on your own reasoning before accepting a claim. Imagining Henry interpersonally, however, quickly reveals just how bad it would be to be him. For example, picture Henry attempting a romantic partnership. Unfortunately, being an extreme doubter, he can’t simply accept his partner’s testimony that she loves him. He doesn’t disbelieve it—he doesn’t believe “it’s false that you love me”—he just doesn’t believe that his partner loves him. Intuitively, however, incessantly doubting “my partner loves me” would be hurtful to one’s partner, everything else being equal (assuming an otherwise good relationship, etc.)

Now consider a variation of Henry’s case, where we focus on his prior first-order evidence bearing on the question “does my partner love me?” And think about how Henry, *qua* doubter, would handle that evidence. Some of this evidence is evidence he got by directly experiencing being with his partner, so surely he wouldn’t just ‘throw away’ that evidence. After all, he satisfies his own way of valuing autonomy, so we might think that he can justifiably believe that his partner loves him.

This is where Henry gets radical, however. He can turn his doubt inward or outward. Going outward is akin to gaslighting. He systematically questions his partner’s experience: “But maybe you are just infatuated. Or maybe you can’t distinguish emotional dependence from love”. Henry’s epistemic insecurity leads his partner to question her sanity. Turning inward, Henry might think: “Maybe the way I remember those events are the result of motivated reasoning. I *want* to believe that my partner loves me, but who am I kidding—wouldn’t I have had similar experiences if this were just infatuation, or if we are *merely* emotionally dependent?” These are not quite the global error possibilities the skeptic employs, but dangerously close. All the same, the extreme doubter doesn’t believe that his partner loves him. Intuitively, this foils his relationship: repeated statements, protestations, and signs to the contrary—“trust me, *I do* love you”—is enough to drive away anyone, and who would blame them?

Here, I am bracketing deeper questions about blame and excuse but it’s natural to think that extreme doubters like Henry are blameworthy for their extreme doubt. Crucially, he is blameworthy for the absence of an attitude—for unbelief (or for failure to manifest a strong enough credence) that is characteristic of someone participating in the kind of intimate practical relationships he’s engaged in. But not all cases are like this. Some people suffer from doubt-obsessive compulsive disorder, on which their extreme doubt is the outgrowth of acute atypical neurology beyond their control. Imagine Henry suffers from doubt-obsessive compulsive disorder, manifesting what clinicians call *obsessional doubt* (Sodré, 1994), pathologically doubting his partner’s love for him. Henry’s condition would be tragic.

We can also think about the way in which a troll might prey on a person’s anxiety. “Why would anyone love you?” says the troll. Understood one way, the troll is inviting Henry to take up the challenge. And anything you do to try to meet it will be met with more suspicion: “Even your parents are faking it”. If one quip doesn’t lead to doubt, the troll will try to undermine your belief with a thousand cuts. Here, we might say that there’s an ethical reason not to engage with the troll’s possibilities; that it’s not only not good for you, but bad for your relationship or even hurtful to engage seriously with the troll’s possibilities. Henry should ignore him.

Finally, consider doubt in the context of rape accusations. As Gardiner (2021) has argued, “many people tend towards a chary ephecticism, viewing withholding as more cautious and virtuous than belief, or they outright disbelieve the accusation” (2022, 393–394). Henry’s boss Billy is accused of raping three low-income women that work for him, all of whom Henry has no independent reason to distrust. Billy’s lawyer argues that Billy has no criminal history, that the women are financially motivated and mutually disliked their boss, which the jurors take seriously enough to consider preponderant, thereby failing to convict him. Now, Henry seems to remember inappropriate behavior from Billy towards the women, but he too fails to believe their accusations: “The jury couldn’t eliminate the possibility that they were lying for money”, he reports. In turn, Henry permanently strains his relationship with them.

What these three cases suggest is that we find it intuitively plausible that engaging in the kind of doubt characteristic of radical skepticism makes it hard to see how we could ethically participate in certain kinds of interpersonal relationships.^10^ Maybe skepticism is practically possible, just not *intimately* practically possible.

One might think that talk of the ‘ethics of skepticism’ is beside the point here because the skeptic ultimately worries that none of our beliefs are justified. Our response should be that if skeptics are concerned with justified belief only, we can question its importance. The reason why is that, if the skeptical problem is worrying only because it threatens the justification of our ordinary beliefs, there remains the question of why we should be worried about the unjustifiedness of those beliefs. A story needs to be told about why a loss of justified ordinary beliefs—‘I have two hands’ and the like—is *really* a bad thing. Why not say: who cares?

Here’s one explanation. Epistemologists often say that truth is the fundamental epistemic good—that truth is what explains the distinctive epistemic goodness of knowledge, justification, and epistemically rational belief—and so one might think that (i) we are missing out on true beliefs if we forgo having any external world beliefs (see Pritchard, 2021) and (ii) this is why it would be bad to be a skeptic.

There are two worries facing this view. The first is that although some epistemologists see truth as intrinsically epistemically valuable—as what anchors the value of knowledge and epistemic rationality—most think that it’s not *that* valuable *qua* intrinsic value (e.g., Sosa, 2003 or that only certain *kinds* of truths matter, i.e., that truth is instrumentally valuable, so that only certain true beliefs matter (DePaul, 2001). So, the reply that we are missing out on true belief if we forgo having any external world beliefs just moves the question: if we lived radical skepticism, and forwent having any external world belief, why care about *that*? Skeptics might add that even though we are missing out on true beliefs, we are also missing out on false beliefs; skeptics avoid both epistemic value *and* disvalue. Skeptics might then say that avoiding the bad is better than getting the good. The skeptic comes out as highly risk-averse. Still, a story will need to be told about why we should follow a norm that systematically leads us to avoid false belief at the cost of any external world belief. And as we will see later, there are significant personal and ethical costs that come with adherence to such a norm. In either case, we have a reply to the purely epistemic view about why skepticism is so alarming.

At any rate, the skeptic doesn’t deny that our beliefs could be true, only that—because of their epistemic unjustifiability—we shouldn’t have any such beliefs. Here, the connection between epistemic justification and normativity might raise eyebrows. “Why think that, from the fact that a belief is unjustified, one shouldn’t hold the belief?” To the *non-normativist* about epistemic justification, who thinks that justification is a non-normative property without any normative oomph, we can say that the burden of argument falls on their shoulders. It is standard epistemic practice to treat epistemic justification as normative. As Parfit urged, we “use the word “irrational” to express a kind of criticism” and the same thought arises for ‘unjustified’ (Parfit, 2011, 33).

Additionally, although few in the debate explicitly construe skepticism as normative, many important thinkers have. Hume’s skeptical worries lead him to say that he was “ready to reject all belief” (Hume *Treatise*, Book I, P.IV, §VII). In contemporary epistemology, Wright (2008) says that the “paradoxes of skepticism … will be adaptable to doxastic norms in general”, that skepticism “discloses a commitment to doxastic norms of warrant”, and that “one should aim wherever possible to have justification and/or reasons for the things one believes” (Wright, 2008, 501–303). Prima facie at least, skepticism is about a thoroughly normative quality.^11^

This pushes the question back to the defender of the threat of skepticism along purely epistemic lines. Suppose the skeptic is right that external world beliefs are all epistemically unjustified. But imagine we hold onto them anyway—and imagine that they’re mostly true! The latter is consistent with skepticism. In this case, it can’t be that we risk missing out on true beliefs. Some *other* story is necessary.

Here’s another possibility. Early on, Barry Stroud (1984) detailed the threat of radical skepticism by drawing our attention to its effect on our relationships with other people:

With respect to what I can know I could not console myself with thoughts of a like‐minded community of perceivers all working together and cheerfully making do with what a communal veil of perception provides. I would have no more reason to believe that there are any other people than I have to believe that I am now sitting in a chair writing (Stroud, 1984, 38).

Eli Hirsch (2018) makes a similar point. He considers the idea that while skeptical problems might lead to the kind of unease some experience when faced with an unsolved logic puzzle, it can produce existential anxiety as well: “If I doubted that I have ever really known anybody, spoken to anybody? If I doubted that I really grew up with my parents and brothers and sisters. Everything that has happened since. My wife, my kids...”, he worries (Hirsch, 2018, 22).

There are two ways to think about these remarks. On a strong reading, it suggests that being concerned about epistemic justification *as such* is itself puzzling. If skeptical doubt reflects a deep existential anxiety, then it’s not about whether our beliefs qualify as epistemically justified simpliciter. Being concerned about whether my belief that (V) “my children’s lives are valuable” or (F) “I have friends” satisfies ‘is justified’ simpliciter just looks like the wrong concern here; it’s certainly not what we would think of as an existential anxiety. On a weaker reading, the existential anxiety reflects broader unease about what a radical disconnect between the mind and world would mean for believers. If your belief that you’re not a brain-in-a-vat is unjustified, this impacts not only your ordinary beliefs, like “the restaurant opens at 12 noon today”, but beliefs that matter to you, like V and F.

Relatedly, Susanna Rinard (2021) says that being a skeptic would not only be “difficult and unpleasant”, but that “it would be deeply depressing to be genuinely uncertain whether your partner, family, and friends exist” (Rinard, 2021, 444). This is why she argues for Pragmatic Skepticism, the conciliatory view that skeptics are right that there’s not sufficient evidence to epistemically justify our beliefs, but wrong to think that we thereby ought to give them up.

Moralists may or may not agree. The *Skeptical Moralist* says that skeptics are technically correct: that, from the ‘epistemic point of view’, we shouldn’t believe (V) or (F), and yet when we consider the ‘ethical point of view’, we are permitted to believe them. Even if skeptics are right in letter, many of our most important beliefs—and their everyday commitments—may remain unperturbed. The *Anti-skeptical Moralist*, by contrast, says that we are permitted to believe (V) or (F), regardless of whether we satisfy the skeptic’s requirements on epistemic justification; the skeptic’s requirements are either no good or can be met in ways that she hasn’t fully appreciated. Whether we are impressed by skeptical epistemology or not, we have added doxastic security: we enjoy the benefits of having a diversity of potentially good epistemic *and* ethical reasons to forego systematic belief-revision. Although both Moralists avoid the doxastically devasting consequence of radical skepticism, they reflect different choice points.

What these observations draw our attention to is that skepticism is worrying partly because it threatens beliefs about other people; crucially, the people that matter to us. This singles out a class of external world belief that we *prima facie* care about. Consider your partner, friend, or family. If skepticism is true, you shouldn’t believe any of them exist. The people that matter most to you would have the same epistemic status for you as hypotheses. This makes the skeptical problem much more pressing. For it highlights how we stand to lose (or never had) something that matters to us. There’s a general point here. The skeptic threatens what we care about. The Moralist develops this insight into an anti-skeptical strategy. Skepticism threatens what matters most to us, and therein lies its weakness. The difficult task is unpacking precisely how to turn this against the skeptic.

Now, notice that the Moralist’s target is *radical skepticism* (or radical skeptical doubt) and not quite the scenario where, say, the Evil Demon Hypothesis is true. The claim is that it would be ethically bad to engage in radical doubt. However, one might think that if the people we love don’t exist—as would be the case, ex hypothesi in the Evil Demon scenario—then you can’t wrong them. But then wouldn’t Moralism only show that it’s wrong to engage in radical doubt *if* external objects really do exist? And, by the skeptic’s lights, aren’t you unjustified in believing that such objects exist, and so aren’t you unjustified in believing that it’s morally wrong to adopt the skeptical attitude?

Although some philosophers think that certain beliefs can wrong just in virtue of what is believed (Basu, 2019a), the Moralist needn’t say (nor deny) that removing certain beliefs can wrong even if the wrong parties don’t exist. Even if it’s true that removing a belief might have wrong-making potential only if the persons for whom *p* refers actually exist, this just qualifies the Moralist’s claim: that provided such persons exist, it can be wrong to lose certain doxastic attitudes bearing on them. In Rinard’s terms, it could be wrong to be “genuinely uncertain” (Rinard, 2021, 444). This normative fact depends on an empirical condition. This is not interestingly different from other anti-skeptical qualifications, however: provided that our senses are reliable, we are epistemically permitted to have external world beliefs, say Reliabilists; provided Evil Demon worlds are remote, we are epistemically permitted to have external world beliefs, say Anti-luck Epistemologists; provided we perceive that we have hands—an empirical claim—our evidence includes the fact that we do, say some Knowledge First epistemologists.

Another way of understanding the Moralist’s claim is that you can’t coherently have interpersonal beliefs of the relevant sort without believing that the relevant agents exist, and so you are committed to the latter by the former. If, however, you doubt that those agents exist, you are thereby committed to doubting your interpersonal claims as well. *This* is what common sense suggests is morally problematic. Radically doubting that your children love you, for example, just seems wrong, and we might use this intuition to push-back on radical skeptical doubt rather than arguing our way out of it using only premises radical skeptics would accept (but more on this in §5). At any rate, the radical skeptic thinks that you shouldn’t believe what you do, independently of the truth of her hypotheses. It’s rather our deficient epistemic relation to skeptical hypotheses which does the heavy lifting. For that reason, engaging with the radical skeptic doesn’t imply engaging with the question: “what should we do if her hypotheses were true?”.

Finally, we should clarify the Moralist’s anti-skeptical goals. Epistemologists typically distinguish between *ambitious* and *modest* anti-skeptical proposals. The former aims to “refute the skeptic on his own terms”, while the latter aims to “diagnose and defuse” radical skepticism, or to “show how to retain as many of our pretheoretical beliefs” as possible (Pryor 2000, 517). Sometimes epistemologists distinguish between the goals of *convincing* the skeptic, *diagnosing* the support for skepticism, and *preventing* skepticism (Williamson 2002, 27),  where the latter can include exposing the flaws in the skeptic’s position as well as methods which pre-empt us from accepting skepticism.

Moralism can be developed ambitiously or modestly. After all, the Moralist can accept the radical skeptic’s claim that our external world beliefs are unjustified. She will then deny that we thereby ought to give them up, since that might be too morally risky, or too severe a eudaimonic cost (§5–6).^12^ This undercuts the doxastic import of radical skepticism. Moreover, the extreme doubter, who embodies the epistemic practices of radical skepticism, is someone who—by the Moralist’s lights at least—incurs serious ethical and personal risks; risks which ought to give anyone attracted to skepticism pause. These risks might lead extreme doubters to reconsider their practices: the potential risks outweigh the attractions. Alternatively, Moralism might convince radical skeptics who otherwise lead ordinary lives to reconsider their position because it would reveal a hitherto unrecognized tension between their position and their conduct.^13^ This has transformative potential; preferences and practices can change once apprised of new information (see Paul, 2014). This doesn’t mean that skeptics would thereby be persuaded into thinking that their position is false, but this possibility can’t be denied either. That a practice is morally wrong is often cited as decisive reason to avoid it. The reflective agent can tolerate practical incongruity for only so long.

Still, the Moralist I explore here is best developed modestly. Skepticism faces an internal difficulty: either giving up beliefs in a way that risks wronging those we care about (§5.2); depriving oneself of the relevant sorts of relationships, a serious limitation; or to continue with them anyway, undermining one’s authenticity (§5.1) In essence, the radical skeptic goes awry in ignoring the ways in which external world belief is tethered to many of our interpersonal practices.

## Skepticism Implies Doxastic Revision

This section argues for the following dilemma. Either skeptical norms of epistemic justification are strongly regulatory of our doxastic economy or they’re not. If so, then they entail that we ought not to have interpersonal beliefs. If they’re not strongly regulatory of our doxastic economy, however, then skepticism is not what I call “agential worrying”—roughly, granting skeptical principles of belief, the failure to meet them would not limit our agency or reduce our ability to exercise our agency; we are rather insulated from any governing influence of those norms or principles.

Our focus is “radical skepticism”. Radical skepticism is typically divided along Cartesian and Pyrrhonian lines, where the former motivates a thesis about the epistemic status of our everyday beliefs, whereas the latter motivates a thesis about propositions which go beyond appearances, namely, that one ought to suspend judgment about them. Cartesian skeptics say that, for any everyday belief that *p*, you lack justification to believe that *p*.^14^ By contrast, Pyrrhonian skeptics say that, for any proposition *p* which goes beyond how things appear to you, you ought to suspend judgment about *p*.^15^

Everyday beliefs are standardly taken to include beliefs like ‘it’s raining’, ‘the door is open’, and the like, while beliefs which go beyond appearances are about how things *are*, not only how they *appear* to be. Now, Cartesian skeptics advocate for the following epistemic principle of belief:

**Cartesian Principle**: You are epistemically justified in believing that P (everyday proposition) only if you are epistemically justified in believing that ~SH (skeptical hypothesis).

Cartesian skeptics argue that you don’t have justification to believe that ~ SH. Together with Cartesian Principle, it follows that you don’t have justification to believe P. And since ‘P’ can be substituted for any everyday proposition, it follows that you don’t have justified everyday beliefs.

What motivates the Cartesian Principle is a closure principle for justification. If you justifiably believe *p* and justifiably believe *q* follows *p*, and deduce *q* as a result, you get justification to believe that *q*. But why think of it as an *epistemic norm*, guiding ordinary belief? One reason is that one rationally ought to conform to it if it is correct. Failing to justifiably deny skeptical hypotheses intuitively implies that one *should not* form everyday beliefs in a guidance-giving sense of ‘should’ (Rinard, 2021). Pausing on this point for a moment, the guidance-giving sense of ‘should’ is one that informs an agent’s cognition and action in cases of deliberation. When you desire to eat another piece of cake but ‘talk yourself out of it’ by thinking “I shouldn’t eat another piece of cake given that I’ve had a large piece already”, the sense in which you ‘should not’ eat the cake is a guidance-giving sense (whatever other senses it might have). You are *pressured* to do (or not to do) something. We can also see this with belief and inference. If you are at a roulette table and have gotten three winning even numbers in a row and find yourself inclined to think that the next winning number will be odd, you might ‘talk yourself out of it’ by thinking “this is a fallacy”. Here too the sense of ‘should-not’ is a sense which captures the pressure *not to do* something. The difference is that while the former informs actions, the latter informs what to think or believe. This doesn’t presuppose pragmatism about belief, the thesis that there are practical reasons for belief, only that *whatever* reasons there are for belief, at least some of them guide what one is to believe. Although this not uncontroversial, I’ll presuppose it here.^16^

While discussion of skepticism tends to focus on its import for everyday belief, it is important to see that the scope of the Cartesian Principle goes far beyond this. It also includes what I’ll call *basic* eros and philia beliefs, like:

Partnership:

Your partner, your companion, or your spouse exists.

Family:

Your siblings, your parents, children, or family exists.

Friendship:

Your friends exist.

Basic eros and philia beliefs are those beliefs which are necessary for the truth and coherence of more fine-grained personal loving-relationship beliefs. For example, I believe that my partner loves me. If I didn’t believe that my partner exists, however, I couldn’t consistently hold that. Indeed, partnership, family, and friendship can express commitments that are integral to who we are.^17^ Revising them can lead us to substantially change who we are or how we understand ourselves, and not merely a shift in the periphery of our web-of-belief. Some might call them local ‘hinges’ against which we make other evaluations (Coliva, 2015; Pritchard, 2015).

There are at least two reasons why basic eros and philia beliefs are within the scope of the Cartesian Principle. The first is that existential propositions about your loved ones are themselves external world propositions. These propositions are about what is there “anyway” (Williams, 2005, 48). They are about mind-independent reality, albeit highly local features of mind-independent reality.^18^ For example, the proposition that:

*Love*: my partner loves me.

Says that there exists an *x* and a *y*, such that *x* is my partner and* y* is me, and that *x* loves *y*. As a result, the existential quantifier ranges over external things—me and my partner—and it says that we stand in a relation—the relation of love. There isn’t any in principle difference between this proposition and the proposition that,

*Hand*: my hand moves across the table.

This proposition says that there exists an *x* and *y*, such that *x* is my hand and* y* the table, and *x* moves across *y*. Here too the existential quantifier ranges over external things—my hand and the table. If existential quantification over external things is sufficient for making P an external world proposition, then both propositions are subject to the Cartesian Principle.

The second reason is that these propositions go beyond appearances (see Greco, 2007). Cartesian skeptics present skeptical hypotheses in which although it *seems* to you that P, possibly ~ P.^19^ The appearance that P is not doubted, only the reality which goes beyond how things appear. It’s easy to see that your basic eros and philia beliefs go beyond appearances too. It’s not simply that you believe that you *seem* to have friends, family, or a partner; you *believe* that you actually do.

The Cartesian Principle’s incursion into the personal doesn’t stop at *basic* eros and philia beliefs, however, but also severs the *personal* eros and philia beliefs you have which presuppose them. For example, consider the following beliefs:

Partnership_PR_:

You are loved by your partner, companion, or spouse.

Family_PR_:

You are loved by your siblings, parents, or other family.

Frienship_PR_:

You are loved by your friends.

If you are in a romantic partnership with someone you sincerely love, you’ll believe partnership. This is not just one belief among many but a conviction you have that you’ve cultivated by engaging intimately with them over time. The same is true for friendship and family.

The trouble is that if you lack justification for basic eros and philia beliefs, it’s hard to see how you could consistently justifiably believe your personal eros and philia beliefs as well, at least where your love is recipient-directed, such that actually existing persons are the target of your attitudes. With the Cartesian Principle in play, we can substitute the everyday proposition *p*—such as that you have hands—with your personal beliefs in two ways, then:

**Cartesian Principle**_**1**_:

If you lack justification to believe ~SH, then you don’t justifiably believe that your friends *exists*.

**Cartesian Principle**_**2**_:

If you lack justification to believe ~SH, then you don’t justifiably believe that your friends *love* you.

The principles thereby range over basic and personal eros and philia beliefs.

What follows for our belief-management if these principles are correct? Epistemic norms would require us to give up these beliefs. And this doesn’t simply imply giving up our *present* beliefs, but to forgo forming new ones. After all, they’re all unjustified—systematically so. The skeptic’s point is not only that you *don’t* have justification for ~ SH, but that you never had it and can’t get it.^20^ Cartesian skepticism is modally strong: it’s about what you can and cannot justifiably believe. So, insofar as the Cartesian Principle is correct and you cannot justifiably deny SH, this supports the following subsidiary epistemic norms:

**Revision Principle:**

You epistemically ought to give up your everyday beliefs.

**Resistance Principle:**

You epistemically ought not form everyday beliefs.

By ‘epistemic ought to give up your belief’, I mean that if you believe that *p*, epistemic rationality implies that you should no longer believe that *p*. The Resistance Principle, in turn, says that you shouldn’t go on to form the belief that *p* either.^21^

Some might see a slight of hand here. Does it follow from the fact that *p* is unjustified for* S* that *S* ought not believe that *p*? The skeptic faces a fork in the road. The skeptic could argue that, although you are not strictly justified in believing that, e.g., you have hands, the fact that it’s unjustified doesn’t mean that you *should* revise your belief; perhaps epistemic justification is not significantly normatively weighty. Alternatively, the skeptic could say that epistemic justification is not normatively weighty at all: it’s merely a kind of non-normative, descriptive property doxastic attitudes might have.

Both responses affect skepticism in interesting ways. The problem is that, if skepticism is best understood as implying that our beliefs are epistemically unjustified, but *not* in a way that reveals any kind of doxastic altering-worthy defect, this would make it less significant from a first-personal point-of-view, where the initial thought was that, *qua* agents, we seek to conform our beliefs to principles of justification and revise them in light of violations, but skeptics have quite literally given us nothing of the sort, even prospectively. Finding out that one doesn’t have a shot at knowing or even so much as justifiably believing that *p* is a rather strong reason to no longer believe that *p*. If, however, someone argues that your beliefs are unjustified, but the quality they are drawing your attention to is one in which its absence doesn’t reflect any significant normative defect, then their challenge is no longer the kind of angst-inducing challenge we originally thought skepticism posed. Indeed, skepticism potentially loses its sting as a philosophical puzzle as well. What grips us is not that our beliefs might lack some non-normative property full-stop, since they unproblematically lack countless non-normative properties already. At best, the non-normative property should be one that *we value *or *have reason to value*. And even if we have reason to value the non-normative property in question, it’s still unclear what would make the skeptical challenge especially distressing. Compare: “your belief might not be true at all worlds”, “your belief might not be empirically verified”, or even “your belief might not be absolutely certain”. Sure, we sometimes have reason to value these properties, but the challenge that maybe our beliefs can’t have them, *despite* being ones that we can appropriately hold anyway, deflates the challenge.

Thus, skepticism would be reduced to a non-existential problem. Moreover, it risks being far less philosophically interesting. Rather, the skeptic seems to think that epistemic justification is normative. Its presence exemplifies something *good*—indeed, something our beliefs ought to have—and its contrary is something *bad*.^22^ However, some epistemologists think of epistemic justification as non-normative; as signaling the fact that the belief fits the evidence, where this fact doesn’t imply that it’s good for doing so, or that it ought to be that way. If they’re right, then the skeptic’s argument about the epistemic justification of external world beliefs is not so interesting—at least for normative epistemology—because it’s a property which tells us nothing about whether it would be good or something we ought to have.

Now, if we understand the radical skeptic as posing an agential worrying challenge, a challenge for how we are to think and what we are to believe, then we should understand the skeptic as a kind of normativist about epistemic justification. So understood, the skeptic’s Resistance and Revision Principles bear on our beliefs. They beget certain subsidiary norms:

**Personal Revision Principle**

You epistemically ought to give up your personal relationship beliefs.

**Personal Resistance Principle:**

You epistemically ought not form personal relationship beliefs.

Given the parity of reasoning made explicit here, then, we can see that if the Cartesian Principle and its unsatisfiability entails that the Revision and Resistance Principles are correct for everyday beliefs, so too it entails that they are correct for your personal (eros and philia) beliefs. In this way, Cartesian skepticism easily intrudes into the personal, on the normative understanding of such skepticism.

Pyrrhonism might have the same implication. Whether it does or not turns on controversial views about how exactly we should interpret Pyrrhonism.^23^ As we’ll see, at least one way of thinking about Pyrrhonism should lead us to assimilate it more closely to Cartesian skepticism, at least in its consequences. Sextus sometimes remarked that Pyrrhonist’s suspend judgment about *all* matters (*PH* I: 31, 205).^24^ If that’s right, then there’s a straightforward implication from Pyrrhonism to suspension of judgment with respect to our personal eros and philia beliefs.^25^

However, Pyrrhonians also deploy the modes for areas such as metaphysics, logic, ethics, and other areas of ‘scientific’ controversy. Indeed, some people doubt the existence of love for psychological and philosophical reasons.^26^ And although judgments like (A) ‘Amit is my friend’ and (B) ‘My child’s life is valuable’ are perfectly ordinary, they are for some philosophically controversial, like (T) ‘There are tables’ or (S) ‘He deserves jailtime’. In this way, while some Pyrrhonians could argue that philia and eros beliefs are outside the scope of suspension proper, but not the other beliefs I cited, they would need to argue that disagreement- and regress-modes that may lead one to suspend on (T) and (S) shouldn’t lead one to doubt (A) or (B). But we might think it’s either all or none of them.

Sextus says that someone “who dogmatizes about a single thing, prefers one appearance to another with respect to credibility, or makes assertions about any non-evident matter, adopts the distinctive character of the dogmatist” (*PH* I 223). So, in reply, someone attracted to Pyrrhonism might think that it’s just *evident* that we have friends, family, or love others, and thus *not* dogmatic that we do.

However, Pyrrhonians think that *p* is non-evident if we balance it by other arguments or perceptions. So, the regress argument can come into play to balance ‘there is no love’ against ‘I love my partner’, or under a certain debunking frame, that perhaps it’s just custom that we think like this. So, Pyrrhonians have the resources to motivate suspension of judgment about these matters.^27^ If that’s right, Pyrrhonism suggests the following anti-eros and philia suspension norms:

**Eros Suspension Norm**:

You ought to suspend judgment about whether anyone loves you.

**Philia Suspension Norm**:

You ought to suspend judgment about whether you have any friends.

We can now see how radical skepticism quite naturally imperils a variety of personal beliefs. Next, we’ll explore how the Moralist uses the skeptic’s strong personal, doxastic regulation to motivate a response.

## The Argument

Where does one go wrong if one follows along faithfully with the extreme doubter, expressing or indeed experiencing the target doubts? The argument goes like this:

(1) If skepticism is true, you epistemically ought to lack intimate interpersonal beliefs.

(2) But it’s prima facie wrong to lack intimate interpersonal belief whilst continuing to engage in intimate interpersonal action or else eudaimonically bad to forego interpersonal action.

Therefore,

(C) If skepticism is true, you either ought to do what’s prima facie wrong or else do something eudaimonically bad, viz., no longer engage in intimate interpersonal action.

The goal of this section is to clarify the premises and forestall certain misunderstandings.

Abstracting from the details, premise 1 says that if condition α obtains (the condition on which skeptical principles are binding on rational agents), then if one has the relevant kinds of interpersonal beliefs B*p*, B*q*, …, one should revise them; specifically, if one believes them, one should do so no longer. This is a point about full belief, but it naturally extends to credences. If one’s credence in *p* is > 0.51, then premise 1 recast says that one’s credence should be below the threshold for belief, or be at whatever imprecise area is sufficient for neither believing *p* nor believing ~ *p*. For ease of exposition, I’ll work with full belief and assume the argument works for credences as well.

Now, one might naturally think that we just can’t shed our beliefs and so it would be problematic if skepticism implied that we ought to do that. The worry here is grounded in an epistemic ‘ought implies can’ principle. Let’s say that’s what you think. Then we can weaken premise 1 as follows:

(1*) If skepticism is true, you should try your best to give up intimate interpersonal belief,

which is compatible with the thought that you cannot (at least directly voluntarily) give up those beliefs. However, epistemologists typically don’t understand ‘can’ so strongly with respect to belief. It’s enough if, as Peels (2017) argues, we can try to influence what we believe. Moreover, the consequent of (1*) be read in different ways. For example:

(1a) You should *try your best to undermine* your intimate interpersonal beliefs, insofar as you find yourself holding them.

(1b) You should *try your best to get yourself in a position where you would give up* intimate interpersonal belief.

(1a) echoes the Pyrrhonian’s recommendation. The Pyrrhonian recognizes that we have a variety of beliefs which make claims on the world, but that we can do things—like employ the modes—to try to undermine them, inspiring suspension. (1b) should be restricted to actions that don’t lead to extreme harms. For example, suicide satisfies (1b) but the thought is that the condition of “getting myself in a position where I would give up belief” may be less extreme than that, akin to Pascal’s recommendation that the non-believer should, failing to believe at will, do what believers do in order to facilitate theistic belief.

Now to Premise 2. Recalling the extreme doubter, the intuition was that he does something wrong, or failing that, something eudaimonically bad. Premise 2 helps to explain why: radical skepticism is the ideal to which the extreme doubter aspires. Were he to succeed, he would compromise the authenticity of his interpersonal relationships, and indeed wrong them, failing to have the fitting doxastic attitudes to his intimates. Otherwise, common sense suggests that foregoing a life of love, friendship, or any sort of commitment-involving intimacy is a serious deprivation. Although some philosophers have suggested that there is something bad about having to give up such personal beliefs, it’s still not clear exactly why this is. Perhaps it’s a starting point for us. As I urged already, common sense suggests as much. But we can do much more than that. In the next section (§5), we’ll see why we should accept this premise. As a preview, doxastic attitudes of the kind the skeptic says we should forgo fulfill what’s necessary for making good on certain interpersonal actions and responsibilities.

## Personal Sacrifice

The Moralist’s second premise is a disjunction. Either it’s morally wrong or else eudaimonically bad to do what skepticism implies. Here, it’s important to understand what this premise means.

For example, one might think it implies—despite ordinary first-order evidence to the contrary—that you should continue to hold your basic interpersonal beliefs come what may. But this is highly suspect, for certainly if your friend asserted “this friendship is over”, then you should *not* continue to hold that they are your friend. Likewise, consider their death. In that case, you shouldn’t believe ‘my friend exists’. Is the main premise of the Moralist’s argument inconsistent with those uncontroversial claims?

Fortunately, that is not how we should understand the premise. The Moralist does not hold that one categorically shouldn’t revise their basic interpersonal beliefs, but that one sometimes can permissibly keep them, *even if* they disrespect certain epistemic norms. Crucially, the Moralist we’re exploring here thinks that, everything else being equal, if revising beliefs risks harm at *t*—of failing in what we owe to the people we love—then one can permissibly retain them *t*. Now, we might add other plausible qualifiers too, such as that it’s morally permissible for one to continue to hold the belief. Here’s an example which illustrates this idea:

*Dissonance*: Amu was recently dumped by Abby, but Amu experiences serious cognitive dissonance. So, she continues to believe that (*L*) *Abby loves her*. Now, imagine believing* L* is strongly beneficial for Amu.

Intuitively, Amu disrespects Abby here. Amu owes it to Abby to treat her as someone whose preferences should be respected, which, we’ll assume, are not for Amu to continue believing that Abby loves her. The Moralist will say believing *L* is impermissible for Amu, and that seems right. But imagine that Abby didn’t care one way or the other. In that case, Amu might *not* be disrespecting Abby. Fortunately, the Moralist is still not committed to saying that it would be permissible for Abby to believe *L*, but she’s also not committed to denying it. It’s interesting to explore this further, but for our purposes the outcome doesn’t matter.

Now, the Moralist argues that skepticism yields highly problematic ethical consequences. This is effectively because of the normative role of interpersonal belief in intimate interpersonal relations. They seem necessary for the authenticity and integrity of love and friendship, for example. This doesn’t mean that such beliefs are necessary *to* love or *to* have friends, but necessary to partake in these relationships authentically or even morally (as we’ll see). This is an attractive premise for the following reasons:

First, when we reflect on cases in which you love someone clearly deserving of love (e.g., your children or best friend), but you consistently suspend judgment about whether you love them (or whether they love you), you mar the relationship. In particular, the relationship is sustained in bad faith. Call this the *authenticity argument*.

Secondly, consider the mutual responsibilities of friendship. Reflection on situations in which a friend is subject to libel or bad-mouthing suggests that you ought to take a stand, in your capacity as their friend, to correct the accusation. Doing so without *believing* the correction, however, would again be in bad faith. And it would, intuitively at least, be a harm to your friend as well. When we consider repugnant trolling, where someone asserts something like “your friends are losers” or “actually, you *don’t* love your children”, or similar, two key ideas stand out. (i) You owe it to them to take a stand—to manifest *belief* that supports them—but epistemic rationality would require that you take no such stand if skepticism held. You intuitively harm them by not doing this, or by suspending. As Basu (2019a, 2019b) has argued, it is “common practice to make claims such as the following: “You shouldn’t have believed that of me”, and that one might be ‘wounded’ by belief (Basu, 2019a, 2019b, 917). The same effect holds for *non-belief*: we can be wounded by non-belief as much as by belief. Indeed, (ii) indecision would reflect a lack of doxastic commitment owed to your significant other. Belief corrects this. Call this the *philial respect argument*. In what follows, we’ll consider these arguments.

### The Authenticity Argument

The authenticity argument presupposes that authenticity is valuable. I don’t defend this here but note that authenticity is widely regarded as integral to one’s flourishing, a virtue, an ethical ideal, and perhaps even necessary for meaning in one’s life. We think it prima facie bad when someone is inauthentic and prima facie good when someone is.

Authenticity can be claimed of artifacts, the self, but also relationships. We’ll focus on the latter two. Authenticity of the self implies ‘being true to oneself’. What does that mean? Guignon (2008) characterizes authencity of the self as follows:

To say that a person is authentic is to say that his or her actions truly express what lies at their origin, that is, the dispositions, feelings, desires, and convictions that motivate them (Guignon, 2008, 278).^28^

Authenticity of the self presupposes that there can be differences or even opposition between one’s actions, avowed preferences, and assertions, with the “core beliefs” that make one who one is (ibid). The authentic person is who one appears to be; the inauthentic person is not. There is a disconnect between self and action. The inauthentic person, then, necessarily engages in practices which misalign with their beliefs, or believes in ways misaligned with their practices. For example, when we think of the person who goes along with the crowd but who does not share the crowd’s beliefs or has no convictions of their own, we think of the inauthentic person.

Inauthenticity of self can of course be blameless. For example, consider Margaret Atwood’s *The Handmaid’s Tale*, a story about a patriarchal totalitarian society which requires women to be ‘handmaids’: slaves who must tragically engage in ceremonial rape. The beliefs of the ‘masters’ and government at large do not align with the main character, Ofred, and the other handmaids. But they are prohibited (for fear of death) from professing their beliefs or to act in accordance them: complicity is demanded. In this way, Ofred leads an inauthentic life but is clearly blameless for it. Still, the inauthenticity of her life is one more bad feature of it.

The Ofred case contrasts with what the Moralist thinks would be true of us were epistemic rationality to demand suspended judgment for our philia and eros beliefs, but we retained our loving relationships anyway. In this scenario, there would be deep misalignment between our actions and our attitudes. We would be blameworthy for leading those relationships inauthentically. This is because we *can* refuse epistemic rationality’s demands in such a scenario; we can quite easily be irrational. Fulfillment of the requirements of rationality, whilst prima facie good, are just not worth the personal sacrifice to one’s loving-relationships—which clearly are worth having, everything else being equal—lest one blameworthily persist inauthentically in those relationships.

It’s important to see that authenticity of self can be had even when one is authentically *bad*. For example, a white supremacist who professes their belief might be authentic but is bad for it. It’s bad for them to have those repugnant beliefs but, crucially, also a harm to others (see Basu, 2019a; 2019b). This is compatible with the idea that being *in*authentic would also be bad for them. Suppression of the self can be prima facie bad for one. Indeed, it can make it harder to overcome doxastic mistakes. For if one’s assertions and presentation of belief are misaligned with their *actual* beliefs, ipso facto one is *not* subjecting their beliefs to public scrutiny. The Millian point intuitively applies here: given the diversity of belief and intellectual skill, the public expression of belief puts one in a better position to become aware of mistakes in their beliefs that might go unnoticed if one kept them private. For this reason, there is a sense in which even the morally repugnant person *is* better-off with authenticity than without. There’s a potential epistemic benefit on the offing; a better chance at epistemic reform, which is harder to see in the case of remaining inauthentic.

The scenario in which one disengages from personal philia and eros belief but nevertheless engages in the relevant kinds of loving-relationships is not like this, however. One maintains an inauthentic relationship and is made worse for it. We can appreciate this by reflecting on two kinds of cases. First:

*Children*. Imagine having children (if you don’t, replace this as necessary—friends, companions, etc). It probably seems to you that they care about you and that you love them. Moreover, you probably believe that you *do* care about them, and that they really *do* care about you. Suppose epistemic rationality demanded that you no longer believe that. In that case, it would still seem to you that they care about you and that you care about them; you would not *stop* caring about them (nor would they stop caring about you).

The phenomenology of care, where there is something that it’s like for you to care for them, to be concerned for their welfare, to love them, and so forth, would not thereby dissipate. But try to imagine the condition you would be in if this combination were realized: that you really don’t believe that they care about you and that you really don’t believe that you care about them. Suppose, for example, that you suspend judgment. If you were epistemically rational vis-à-vis the Revision and Resistance skeptical norms, *that’s* the condition you would realize. This could spoil the relationships. For starters, non-belief might easily spillover into actions that matter for the relationships. In an effort to avoid doxastic and practical fragmentation, your relation to your loved ones might take on a fundamentally different character (e.g., viewing them like anyone else). What’s more, the authenticity of your relationships would be jeopardized. Skeptical norms so understood would demand that you don’t take a stand on whether you love them and whether they love you *because* you would be required *not* to believe either way. In this way, while you would continue to experience the phenomenology of care and participate in those activities tantamount to caring for them, it would be in bad-faith. The intuition here is not just that the skeptic’s ‘neutrality’ is hurtful when made explicit but that their exercise of agency is objectionable or even blameworthy: it is blameworthy to feign belief here in tandem with actions characteristic of love and otherwise unsettling to harbor such doxastic indecision with firm practical commitments. Next:

*Priest*. Imagine the priest who, having lost his faith fully and is now a committed agnostic retains his priesthood and continues his sermons as before. Imagine that his congregation would, quite naturally, prefer him to be faithful but certainly not to continue in his religious role unchanged whilst being a committed agnostic.

The priest’s relationship with his congregation would be in bad faith. This is not a praiseworthy way to live; one is quite truly at odds with oneself.

Now, this doesn’t mean that we can’t sometimes admire people for their uncertainty. Many admirable people are committed to long-term projects whilst intermittently lapsing in certain convictions tantamount to authentic participation in their projects. The point is rather that someone who *systematically* feigns belief through practice or otherwise lacks belief characteristic of the person committed to a project is a kind of fraud, a mere phony. The argument is that the *Priest* and *Children* cases are structurally analogous to what would be so if one respected epistemic rationality as the skeptic understands it, eliminating the relevant beliefs in turn, but continued to maintain their personal relationships anyway. It would be in bad faith, and thereby inauthentic. Insofar as authenticity is desirable and inauthenticity avoidable, it should be avoided.

One could push back here and say that experiencing the phenomenology of care—rather than belief, or some sort of strong doxastic commitment (e.g., > 0.7 credence)—is all that’s necessary for authentic and respectful participation in personal loving-relationships. On this view, if one suspended judgment about whether they love their spouse and whether their spouse loved them, for example, but still experienced care for them, there would be no inauthenticity, no bad-faith, and no lack of respect.

But this objection really stretches the weight we attach to doxastic commitment in personal relationships. It’s hard to see how a spouse might be said to authentically partake in love with her partner if, for example, she’s not even remotely convinced that her partner loves her, nor that she loves her partner. Imagine for a moment if your partner, parent, or best friend consistently suspended judgment about whether you love them, on whether they’re your friend, or you’re they’re friend, or on whether you have any value, and so forth. Credence above suspension, or outright belief, settles these questions whereas the phenomenology of care alone doesn’t manifest any commitment—and might systematically co-exist with genuine doubts, the longing to be without them, or even ambivalence towards the person.

One might also say that we’re not making the relevant contrast. The relevant contrast is the possibility that the loved one doesn’t exist, since they fall within the scope of radical skeptical doubt. It might be weird for a loved one to doubt that I exist, but is it hurtful or disrespectful to doubt my existence if it follows from their general doubt about the external world?^29^ I think so. What we have put our finger on here is that there’s something ethically worrying about *radical skeptical doubts*. Doubting the existence of your children commits you to doubting that they love you too. The skeptic demands you to do the former but conceals in that requirement a terrible commitment. One should take ownership of their commitments.

Although as philosophers who take seriously what others wouldn’t, we might struggle to see why it is hurtful to consistently engage in radical skeptical doubt. But imagine telling a non-philosopher intimate that you really do doubt their love, their thoughts, their intentions, and so forth because you really doubt other minds, the external world, and so forth. Aside from worrying that you might be experiencing a psychotic break from reality, they’d likely be hurt by the suggestion that you doubt them because of *skeptical arguments*. “What kind of person are you?”, they might wonder. Obviously if you are unmoved by this intuition, it may be difficult to get you into the mindset whereby it becomes more plausible. What we can draw our attention to is that the philosophical source—skeptical arguments—for radical doubt has terrible doxastic consequences, so much so that one can be ethically evaluated for it, just as we might ethically evaluate an agent’s intention to do what’s right under the guise of the good when it is actually radically misaligned with what’s good. We might still judge them as awful for their actions (or their consequences) committed in the name of the good. That their intentions flow from their more general goal of ‘doing what’s good’ doesn’t do much to ameliorate the sense of wrongness we experience. Similarly, that the skeptic’s intention is to ‘doubt external reality, other minds’, and so forth, needn’t stop us from ethically evaluating their commitments (or their consequences); it needn’t ameliorate the sense of wrongness we experience to be on the receiving end of the kind of detached doubt of the ‘pure inquirer’ (see Williams, 2005). More generally, we can say that the pure inquirer—taken to the limits of radical skepticism—is hurtful.

### The Philial Respect Argument

We owe our friends certain things in virtue of being their friends.^30^ A plausible idea is that we owe our friends *recognition*: that we recognize our friends as such; that we properly regard them as our friends when friendship-making conditions obtain. The same applies, *mutatis mutandis*, to other persons with which we share personal loving-relationships. We owe our partner or spouse certain things in virtue of being their partner. We should regard them as loving us and ourselves as loving them (provided that we do). We can also see this as a precondition for more robust obligations, such as trust and mutual self-disclosure (Annas, 1988; Alfano, 2016; Thomas, 2013).

The claim here is not that to *be* a friend or to *be* a partner one must have these beliefs but that being a *good* friend or a *good* partner requires one to have these beliefs, where ‘being a good friend/partner’ entails that one does not consistently do wrong by them. Of course, we sometimes do harm our friends and remain good friends nonetheless, but what being a good friend requires is the avoidance of systematic harm. The problem is that to fail systematically in one’s friendship responsibilities *is* consistently a harm to one’s friend. The argument from philial respect maintains that to consistently suspend judgment on the most basic philia and eros propositions about who we love is such a failure.

This argument clearly presupposes three theses: first, that there are philial responsibilities; second, that one of them is that we do not refrain, at least consistently, from having basic and certain personal philial beliefs; and, third and less obviously, that flouting one’s philial responsibilities are sometimes a serious harm to one’s friends or other loved-ones. The Moralist therefore needs to say something in favor of these ideas.

Philial responsibilities are widely recognized in moral philosophy (Helm, 2017). Wallace (2012) thinks that they are a species of more a general phenomenon, ‘duties of love’, which we owe to persons with whom we stand in personal loving-relationships. And while many philosophers recognize friendship, parenting, and companionship as involving *special obligations*, the Moralist only needs to lean on the idea that these relationships imply mutual responsibilities and not agent-relative reasons over and above moral reasons. I’ll presuppose that personal loving-relationships imply mutual responsibilities, but the crucial question is whether this includes the duty to believe of our friends (etc.) that they are our friends (etc.), and that we bear the relevant attitudes to each other once we partake in these relationships overtime.^31^ This seems intuitively correct for *good* friendship (etc.). To see why, consider:

*Beliefless Friendship*: Li has been friends with Lo all his life, and practically treats him as a best friend—routinely enjoying their time together, frequently speaking with him, thinking fondly of him, and so forth. However, Li isn’t decided about whether Lo even exists. “For all I can tell, you’re just a figment of my imagination”, he thinks without ever disclosing.

Intuitively, Li is failing in his capacity as a friend here. This is readily seen by the fact that Li conceals his indecision. Imagine if he were explicit: “Look, Lo, I’m actually *not* sure that we’ve been friends all these years. I’m not sure that you even *exist*” and “For all that I can tell, you don’t care about me. It might *seem* that way to me, but I’m not committal one way or the other”. Here, Li is disrespectful. He wrongs his friend in his capacity as his friend. It’s a *philial* responsibility because it’s not so clear that Li wrongs people he doesn’t know by taking an indecision attitude towards *their* existence or care. It might be strange or impolite, but not clearly wrong. Not so in the case of friendship, partnership, parenting, or other close ties.^32^

A final consideration is that it would be *emotional free-riding*. Although one might have the fitting emotional profile to their loved-ones, they don’t have any of the *doxastic commitments* which manifest taking a stand on their relationships. To see why, suppose someone said to you: “Your children’s lives are not worth living”, “You don’t really love them”, or “They shouldn’t have been born” (*mutatis mutandis* for the fitting personal loving-relationship). What would the skeptic’s epistemic norms require of you here? It requires you to *refrain* from believing ‘No, my children’s lives *are* worth living’, ‘No, I *do* love them’, etc., and thereby to forego taking a stand which supports them—to stand *for* them.^33^ As a parent, not only does it seem permissible to maintain those beliefs, but you really ought to do so. Likewise for a partner or friend. For what kind of parent, partner, or friend would one be that *didn’t* do this? Our philia responsibilities say that we ought to take a stand, but the skeptic forbids those beliefs which manifest our doxastic commitments to others. In this way, being a skeptic would lead us to be morally bad agents.

## Clarifications

This section clarifies what Moralism implies, highlighting its relationship to other views in the literature, and details how it interacts with the radical skeptical paradox.

As I mentioned earlier, there are other contemporary Moralist-like positions. For example, Hirsch (2018) has recently presented two arguments for the conclusion that no one can responsibly “doubt external reality” (Hirsch, 2018, 155). There are important analogies as well as disanalogies between my arguments and Hirsh’s “argument from valuing” and his “loss of self” arguments. The argument from valuing is that if one hasn’t interacted with other people, one doesn’t value their own life, and that one couldn’t be intellectually responsible if one didn’t value anything in their life (Hirsch, 2018, 155). The premises link valuing one’s life with interpersonal relationships as well as intellectual responsibility with value. The argument from loss-of-self takes the starting point of solipsism and tries to determine whether one could maintain a coherent sense of self over time, but argues that it’s impossible. The key premises are that it’s impossible for one to have what Hirsch calls “self-esteem” if one doesn’t believe that one has interacted with other people; that it’s impossible to have a *self* without self-esteem; and that one cannot be intellectually responsible without having a self (Hirsch, 2018, 179). The point of intersection is that we both appeal to what makes life go well in response to skepticism.

Hirsch’s Moralist (Lev) departs from our own, however. The key points of our argument are that skeptical norms imply that we should refrain from interpersonal believing, but that this seems to be morally wrong or else eudaimonically bad. What we should draw from this with respect to skepticism’s tenability or correctness is a further issue (one we’ll come to in a moment). Hirsch’s Moralist (Lev) seeks a much stronger conclusion, that “certain doubts are impossible, not as a matter of contingent psychology, but as a matter of necessity”, a much stronger view than ours, which is the conditional about the ethics of skepticism (Hirsch, 2018, 152).

The other reason is that the premises of Hirsch’s argument appear stronger than ours too. Hirsch tells us that: “it is impossible for a being to value its life, or anything in its life, if it does not believe it has meaningfully interacted with other lives” (ibid, 155). This is a strong view about the requirements of valuing one’s life. By contrast, the key premise of our argument appeals to familiar ideas about love and friendship: that being a good friend or partner means having certain beliefs about one’s friend or partner; and that one can be wrongful towards the people with whom one shares personal loving-relationships by not having certain attitudes about them. Although the *arguments* I present in favor of these ideas are not commonplace, the *ideas* themselves are.

Some say that radical skepticism is best understood not a position but as a *paradox*, one which reveals a tension between intuitive epistemological ideas (Pritchard, 2005; Wright, 1991). Moreover, the goal of any anti-skeptical strategy ought to be to explain why the skeptic’s reasoning seemed compelling, but is nevertheless mistaken. Since the Moralist is treating radical skepticism as a position, however, we might worry that it isn’t a satisfactory anti-skeptical strategy.

I’ve only argued that skepticism yields a kind of first-personal ethical dilemma: to either abandon interpersonal beliefs—which intuitively will be wrongful—or else, in an effort to maintain authenticity and an unfragmented self, to forego the relevant interpersonal relations, securing a kind of deprivation. But maybe you want more from Moralism than that, so we’ll explore that possibility here.

The Abrogation Moralist can agree with the skeptic’s epistemology. Maybe the skeptical reasoning looks compelling because it *is* compelling, and the Abrogationist can accept that, but this doesn’t mean that we should thereby give up our interpersonal beliefs. Perhaps they are prudentially justified, and their prudential justification is enough to hold them *despite* their epistemic defects. Here, the Abrogationist could argue that the epistemic-ought, to the extent that it is guidance-giving, is not *authoritative*. Since living without interpersonal belief while engaging in the relevant interpersonal relations would be wrong and inauthentic, and otherwise forgoing those relations would be a serious deprivation, the prudential costs here should not be divorced from our deliberations.

To clarify, the Abrogationist is not committed to saying that the epistemic-ought is *not* guidance-giving, only that the pressure exerted by it (if any in the case) doesn’t overcome the pressure exerted by the ethical reasons to maintain belief. One really ought to retain their prior beliefs and so one really ought to ignore the demands of the skeptical epistemic norms in play here.^34^ The skeptic argues that you epistemically ought to give up external world belief, but she fails to see that the epistemic ought just shouldn’t always move one doxastically.

A second clarification: The Abrogationist doesn’t need to say that ethical reasons are *always* more authoritative than epistemic reasons, but she doesn’t need to deny it either. What she holds is that these reasons in fact dominate in these cases because there are weightier normative considerations. Put another way, the guidance-givingness of the epistemic-ought is obstructed by the agent’s concern for moral and prudential goods. Skepticism is effectively idle, even though it aspires to guide the intellect.

The Encroachment Moralist, by contrast, tells us that the ethical features of believing that *p* affects its epistemic status. So, while the skeptic says that *p* can’t be justified, the Encroachment Moralist will deny this. In particular, she’ll take issue with the premise that you cannot justifiably believe that ~ BIV as follows: either skeptical hypotheses *are* excluded as error-possibilities, owing to their risky ethically deleterious effects, or else they *can* be eliminated, in a Moorean-way, owing to the weakened evidential standards for justification.

How might this work? Consider an agent’s personal experiences with their children, partner, or friends, which supports believing some everyday *p* (e.g., “I met my friend at a café today”). These experiences don’t favor ~ BIV, to be sure. They don’t tell us anything about whether BIV holds. But crucially, that isn’t necessary because of the ethical risks with considering the BIV scenario seriously. Considering that scenario seriously—as a live hypothesis about what one’s life might be like, whereby it is something one must eliminate by way of epistemically non-circular reasons—would mean *bracketing* your personal experiences with your loved ones, their testimony, what you believe about them, your shared memories, and so forth, as the sorts of reasons you may employ to counter the BIV hypothesis. Even this kind of evidential bracketing seems morally suspicious.

Interestingly, the encroachment mechanism here is different from standard moral encroachment cases. In those cases, the moral risk of error makes certain possibilities relevant: that the black man in black-tie at an affluent party might be a patron *is* a relevant alternative, given the moral risk of forming racist stereotype beliefs, despite the likelihood that he is an employee (see Gardiner, 2018). In our *eros* and *philial* cases, however, the encroachment mechanism is inverted: certain possibilities are irrelevant or subject to lower standards because of the moral risk they pose to what one already believes. The more one considers skeptical hypotheses, the more one exerts pressure on beliefs which are tantamount to not being disrespectful, hurtful, or inauthentic. This can make the hypotheses subject to lower standards for rational rejection than hypotheses which are purely theoretical or lack any clear value-reducing impact. The result is that from one’s belief that *p*, one can competently deduce that ~ BIV, given the prior support for their belief. What made the skeptical reasoning look plausible is the presupposition that epistemic justification is *pure*, when in fact it is affected by ethical factors. In either case, the Moralist can explain why the skeptical reasoning looked plausible *and* why it is defective.

But how does this encroachment mechanism work, more exactly? We’ll pause here to consider how moral encroachment is supported and illustrate the comparison. Moss (2018), for instance, argues for moral encroachment by way of its explanatory power. You see a dog-walker and their pit-bull approaching you in the evening, and you cross the street to avoid them. You believe, given the statistical evidence, that (I) “The dog is more likely to bite me than the other dogs”. If someone challenged you, you’d be justified in asserting (I), says Moss. She contrasts this with a racial profiling case. Suppose you’re in a city and a hooded black man is approaching you in the evening. You cross the street to avoid them. You believe, given the statistical evidence, that (II) “The person is more likely to rob me than the others”. Moss says that (II) is unjustified. The epistemic difference is explained by moral encroachment:

The pedestrian uses statistical evidence to form an opinion about a pit bull and also to form an opinion about a person. The former opinion is knowledge and the latter is not. The moral encroachment thesis accounts for this contrast, by allowing that the moral status of a profiled object can make a difference to the epistemic features of opinions that are formed by profiling it (Moss, 2018, 180).

Crucially, Moss says that the problem is not with the epistemic deficiencies of statistical evidence as such, but with the *moral risk* of racism posed by using the (otherwise good) evidence one has for (II). Arguably, the example should hold for other moral risks as well, adapting the case to fit.

Here, the Moralist can make a similar move. Ordinarily, the evidence we have for believing (I*) “Here’s a table” is not sufficient for rejecting the BIV hypothesis (Rinard, 2021; Silins, 2007). One reason is that our evidence seems ‘epistemically circular’, in the sense that you already need to take for granted that the BIV hypothesis is false for your ordinary sensory, memorial, or testimonial evidence to have that kind of anti-skeptical purport (Coliva, 2015; Wright, 2014). Another reason is that it’s the same evidence we’d have whether BIV holds (Brueckner, 2005; Walker, 2015; Nozick, 1981). Finally, one might think that ordinary evidence doesn’t carry any information about the BIV hypothesis’s likelihood. This, in effect, is what Dretske (2005) and Wright (2004) have argued: that there’s a lack of evidential transmission between ordinary evidence and ‘heavyweight’ hypotheses, like the BIV and Evil demon hypotheses.^35^ It will take us too far afield to critically explore these views here; it’s enough to say that the Moralist could grant that our ordinary evidence doesn’t favor rejecting skeptical hypotheses.

Now imagine the skeptic raises the possibility that you don’t love your children—that love is merely an appearance—that your friends are always ‘faking it’, or even that you or others you care most about aren’t worthy of care; that appearances might be radically misleading. “How?”, you ask. She says that maybe you are manipulated by Descartes’ Evil demon, or that perhaps you’re alone experiencing a vivid simulated world as a BIV. Here, you immediately see the moral risk: “That’s a terrible thing for me to think about my friends. Surely (II*) they do care for me”, or (II**) “I do love my children, and they care for me as well”. As we explored in §5, the ethical risks are grounded in what we owe them: respect, harm avoidance, and authenticity, but it’s enough to see the intuitive sense that there’s something morally depraved or at least morally ambivalent about considering seriously the radical skeptic’s hypotheses. (II*), of course, commits you to the existence of your friends, and likewise (II**) to your children, etc. So, you’re committed to other minds. But you’re also, given the argument from §1, committed to external things.^36^

Still, you might worry that this is a pyrrhic victory. The Moralist saves our personal beliefs, but what about the rest? There are at least two responses. First, moralism can be seen as a via* media* between anti-skepticism and skepticism. After all, skeptical reasoning seems intuitively compelling, and perhaps it is correct—only epistemically. Again, the Moralist might say that the skeptic overestimates the oomph of the epistemic-ought, however, and thus her principles may not have the suasive force she thinks they do. This makes Moralism like Rinard’s (2022) Pragmatic Skeptic, but importantly weaker. The pragmatic skepticism Rinard defends says that (i) that “we lack evidence for ordinary beliefs”; that (ii) “there are only practical reasons for belief”, and (iii) “we ought to retain these [external world] beliefs, because we are all better off doing so” (Rinard, 2022, 436). Our stronger Moralist accepts (i) and (iii) but crucially denies (ii). There are epistemic reasons for belief too, it’s just that our ethical reasons are sometimes *good enough* for belief retention. In effect, Moralism gives us a way of explaining the intuitiveness of skeptical reasoning without losing many of the beliefs that matter most to us.

The second response is that the Moralist needn’t concede so much. To see why, consider the following closure principle:

**Practical Closure**: If S is practically justified in believing that P, and if S believes (or ought to believe) if P → Q, then S is practically justified in believing that Q.

If practical closure holds, many of our beliefs would fall outside skeptic’s reach. If, from a purely practical point of view, you ought to believe ‘my children exist’, it follows that you ought to believe that there is an external world and other minds, since they are recognized implications of the relevant beliefs, at least understood conservatively.^37^ So, there would be fewer limitations on the Moralist’s response than it initially appeared. To be sure, this wouldn’t rescue them *epistemically*, but you would still be permitted to believe them, which is precisely what the strong Moralist hopes for.

We’ve seen how Moralism can be developed as an anti-skeptical theory. But what good is Moralism if theorists already have an anti-skeptical theory; why one more theory on the market? Here’s why: when dealing with morally and personally risky positions like radical skepticism, theorists should want all the protection they can get—the more the merrier. Put generally, a diversity of anti-skeptical reasons is doxastically safer than only one kind as it makes one less vulnerable to having their beliefs undermined.^38^

Another point is that Moralism benefits other anti-skeptics. It will take us too far afield to explore this in detail, but ethical and epistemic responses to skepticism can be mutually supportive. If some anti-skeptical epistemology works, then Moralism plus that anti-skeptical epistemology gives us a diversity of reasons for resisting skepticism, which further protects our beliefs for skeptical criticism. We want security for our beliefs. And if the epistemology fails, we still have sufficient reasons for resisting skepticism. Either way, the ethical and epistemic strategies work in tandem to safeguard our beliefs and the practices which involve them. It’s far more beneficial dialectically to have many kinds of responses to powerful arguments than only one kind. As we saw, Moralism harmonizes with certain epistemological anti-skeptical theories, but it also works as a general buffer for our beliefs from skeptical criticism. In this way, Moralism isn’t only additional immunization against skepticism.

Moreover, consider again the Abrogationist development of Moralism, which is strictly compatible with the letter of skepticism: it could be that we are epistemically unjustified in believing what we do, it’s just that we lack sufficient reason to abandon our beliefs given the moral and personal costs of doing so.^39^ Hence, to the question “what good is Moralism qua anti-skeptical theory?”, the Abrogationist version of Moralism says: we don’t even need anti-skeptical epistemology, since Moralism robs skepticism of its doxastic potential.

Finally, since radical skeptics are committed to radical doubt, then wouldn’t they also resist the claim that their skeptical ways are at odds with an ethical life, i.e., wouldn’t skeptics radically doubt those claims too? Not necessarily. The skeptic is committed to thinking that external world beliefs are unjustified, and not necessarily that other, non-external world beliefs are unjustified. The claims I argued for to support Moralism (§5) seem entirely priori, flowing from armchair reflection. It’s one thing to doubt the premises of the arguments for those claims because there are better alternatives in meta-ethical theory, and quite another to doubt them owing to radical skepticism. If *that* were the skeptic’s position, she’s no longer only a radical external world skeptic, but a sort of a priori skeptic or even a meta-ethical skeptic about prudential reasons, the good life, or moral risk. Hence, the Moralist’s dilemma might further ‘radicalize’ the radical skeptic.

Another option is that radical skeptics might face the dilemma I presented head on, choosing between embracing the moral criticism we set against them (akin to adopting egoism—e.g., “It doesn’t matter to me that I wrong those I am personally related to”) and serious self-deprivation (akin to adopting asceticism—e.g., “I want to live without personal relationships”). The radical skeptic would need to argue that they are costs worth embracing. The Moralist councils us against pursing those options. Still, the Moralist’s conditional criticism remains: if skeptics are right, they need to choose between a morally objectionable or personally impoverished life. A skeptic who sits-on-the-fence, who thinks that they can lead an appealing life continuous with their radical doubt might be motivated to reconsider.

## Tranquility?

Pyrrhonians say that suspension of judgment leads to *ataraxia*; ‘tranquility’ or ‘undisturbedness’, and in this way contributes to one’s flourishing. So, is the Moralist right to think that certain cases of suspension of judgment are detrimental to one’s flourishing? If so, isn’t the Moralist at odds not only with Pyrrhonism as a *skeptical ideal*, but also as a *good way of life*? Even if the Moralist succeeds against the first sort of Pyrrhonian, why think she succeeds against the second sort? As Annas and Barnes put it: “we should read Sextus in order to become happy” (Annas & Barnes, 2000, xxx). Therefore, the Moralist needs to argue that Pyrrhonism is wrong about the good life too.

Pyrrhonians lack beliefs which go beyond appearances. They avoid forming those kinds of beliefs because “all unhappiness occurs because of some disturbance”, and arguments for and against positions which go beyond appearances cause disturbance (AD 5.112). I’ve argued that the same is true of interpersonal relationships (§2), but the goal is not simply to make explicit what Pyrrhonians are committed to, but why it is ethically problematic to be committed to these cases of suspension of judgement. The objection is that the Pyrrhonian is concerned with living the good life too and at least acts as if suspending judgment is conducive to that end. The character with which the Moralist is engaging is the Pyrrhonian who acts as if suspension of judgment is conducive to their flourishing, but of course doesn’t *believe* that it is. The Moralist is not saying that the Pyrrhonian won’t be tranquil by suspending judgment, but that suspension of judgment about the personal seems to do wrong to those with whom one is intimately related. This is what makes the Moralist different from other critics of Pyrrhonism. In what follows, it will be helpful to contrast the Moralist with those criticisms and then explain how the Moralist provides reasons for thinking that the Pyrrhonist is wrong about the good life.

For example, Mates (1996) has argued that disagreements can be exciting and challenging. In this way, it can be pleasurable for one to maintain belief in the face of controversy (Mates, 1996, 75–77). Annas (1988) argues that belief in objective moral rightness and wrongness can make one feel more secure in one’s actions; the lack of that security can cause distress or anxiety, which is intuitively at odds with one’s flourishing.

As Machuca argues, the Pyrrhonian replies that these are all contingent features of one’s psychology (Machuca, 2019, 44). Some experience pleasure from maintaining disagreement; others not. Some people experience security in having beliefs about what is right or wrong. Pyrrhonians report disturbance. However, both Pyrrhonians *and* Annas and Mates can be right here. This is easier to appreciate once we see that subjective theories of flourishing are implicit in this dispute, whereby one flourishes in one’s life only if one’s desires are satisfied, one’s preferences are fulfilled, or one has pleasurable experiences.^40^ Now suppose that, as a Pyrrhonian, one desires an undisturbed life and adhering to Pyrrhonian norms leads to the fulfillment of that desire. Of course, *some* people will desire excitement and doxastic security instead. Conditional on those preferences, adhering to the Pyrrhonian norms would *not* lead to the fulfillment of their desires.

The Moralist, by contrast, can say two things here. First, flourishing plausibly goes beyond the fulfillment of desires and preference-satisfaction, thereby avoiding the objection—which might be successful in the case of Annas and Mates—that whether suspension of judgment is ethically bad for one depends on “one’s personality or temperament” (Machuca, 2019, 50). This is because the Moralist is making an a priori claim about what is good *in addition to* the satisfaction of one’s desires, preferences, or experiences. This doesn’t entail that experience, desire- or preference-satisfaction are ethically irrelevant, only that they don’t have a monopoly on the good life.

Second, if the Pyrrhonian suspends judgment about their loved ones—all the while persisting in their interpersonal relationships—they are doing something morally wrong to their intimates. It doesn’t matter that they *also* suspend judgment about whether it is morally wrong as well. Morality doesn’t depend on individual judgment in that way (if at all).

Finally, insofar as Pyrrhonians forego such interpersonal relationships, owing to the potential wrongs that come from the lack of fitting beliefs towards one’s loved ones, they are thereby depriving themselves; it is a severe kind of asceticism. Although some might say that genuine deprivation requires desire—and that Pyrrhonians could lack the requisite desires—obviously this is a contingent matter; some attracted to Pyrrhonism might also consistently desire interpersonal relationships but forego them in an effort to avoid moral risk. They might also accept such desire-frustration as costs worth incurring. Then the Moralist’s complaint reveals that Pyrrhonians must make a serious choice: either a severely ascetic lifestyle, or else jeopardize their morality and authenticity. Again, being with your partner, say, all the while systematically suspending judgment about whether they love you, care about you, or whether they even exist *seems wrong*; like a personal failure as their partner. And, if to avoid this condition, you systematically forego such personal relationships, isn’t that a serious deprivation? Even if we find Pyrrhonians who embrace the ascetic life as admirable, the Moralist’s point is only that Pyrrhonians cannot avoid asceticism or else blameworthy practices.

## Conclusion

Moralism has given us new resources for resisting radical skepticism. The skeptic faces a serious dilemma about how to live. The doubt characteristic of radical skepticism presents considerable ethical and personal costs. Although much more can be said than I have defended here, my primary goal was exploratory and explanatory: to explore why Moralism is a plausible anti-skeptical strategy and to pivot our attention to the *ethics of skepticism.*

Moralism has also provided us with a potential *via media* between traditional anti-skeptical proposals and skepticism. Looking ahead, we might think that Moralism shouldn’t stop with our intimate personal beliefs. Instead, certain basic moral and political beliefs, or indeed beliefs that are essential to our identities, essential for what Williams (1973) called our ‘ground projects’, carve out spaces for us to lead meaningful lives reflective of who we are, ones that are not to be so easily abandoned.

Finally, many of our political beliefs are expressions of what we morally or politically owe to others as matters of justice. Utilizing skeptical arguments to motivate suspending judgment about whether, say, there is institutional racism in the United States seems wrong. Likewise, systematic suspension of judgment about endemic sexism or racism seems wrong. We might also worry that suspending judgment about the suffering of the many millions of people in the world is dehumanizing.^41^ Giving up our beliefs—as skeptics would recommend—jeopardizes our potential for greater social and political justice. Indeed, it is disrespectful to the victims of injustice. I think that’s right, but here I have only explored one way of developing Moralism.^42^

## Footnotes

1 This doesn’t mean that the skeptic isn’t making an epistemic mistake. §6 explores how ethical and epistemic criticisms of radical skepticism might harmonize.

2 More specifically, Cartesian skeptics say this. Other skeptics, like Pyrrhonians, might appeal to the regress argument or to widespread disagreement. For example, Cling (2009) argues that we may have to accept “in a tragic spirit” that we lack adequate epistemic reasons for what we believe (2009, 340). Others, like Machuca (2019), might argue that due to expert disagreement about the nature of love, we ought to suspend judgment about the matter, since even considerations of self-trust or symmetry breakers will also lead to disagreements about the disagreement that exert reasons to suspend.

3 Moralism is similar to the apraxia objection, on which skeptics cannot act or act rationally. Moralists diverge from the proponents of the apraxia objection by highlighting how even if the skeptic can act, or rationally act, they can’t do so without incurring serious personal and ethical costs. Even if there is no deep incongruity between systematic non-belief or suspension of judgment and rational action, skeptics face a costly ethical dilemma. Similarly, Bett (2010) argues that it’s hard to see how Pyrrhonians could be reformers, which is a kind of normative criticism. Historical figures close to Moralism include Cicero and Aristocles (albeit the latter more about consistently obeying law).

4 To clarify, the principal target is the radical skeptic: someone who thinks that we systematically lack justification for our external world beliefs. It is helpful to distinguish them from those who only ‘regard it as defensible’ (see Goldberg 2013). A philosopher might professionally engage in the practice of defending radical skepticism, but not *believe* or *accept* it. Moralism has persuasion potential here. After all, why continue to regard radical skepticism as defensible if it’s ethically indefensible?

5 A quick note about terminology. By ‘morally wrong’, I have common sense morality in mind: what is wrongful by ordinary standards. By ‘eudaimonially bad’, I mean what, again by common sense standards, would make one’s life worse or curtail living well: consistent tragedy or suffering, undesired deprivations of goods, etc.

6 Other philosophers mean something more comprehensive, like reasons which count towards making oneself better-off, or reasons that are either prudential or moral (Leary 2017; Rinard 2021).

7 Many profess attraction to skepticism or even defend it. See Stroud (1984), Rinard (2017a, 2017b), Pritchard (2005), Fumerton (2005), and Brueckner (2014).

8 See Prichard (2015).

9 Contemporary anti-skeptical epistemology since the 1980’s has decidedly been unambitious; it doesn’t try to convince the skeptic. See, e.g., Nozick (1981), Pryor (2000), and Williams (1996) , and Williamson (2002). 

10 To clarify further, the claim is not that, if Henry has evidence to the contrary—testimony from his partner, or experiences which are suggestive of waning love—that he should still not suspend judgment about whether his partner loves him. Rather, the claim is about how Henry should engage with radical skeptics and their brethren ‘in the wild’, like the troll. The radical skeptic argues that even though it seems to Henry that he is loved, it’s not good enough for believing that he is because he cannot justifiably exclude the radical skeptic’s hypothesis. However, Henry would be morally praiseworthy for ignoring the troll’s hypothesis. However, there is a potential stalker problem here. Consider Henry*, who stalks a celebrity, convinced that the celebrity loves him, despite clear protestations that suggest otherwise. Wouldn’t Henry* see the celebrity the way that Henry sees the troll? In both cases, the agent has appearances which suggest that *p*, but in one case it seems right for the agent to ignore ~ *p* counter-possibilities (the troll’s) but not the other (the celebrity’s). Stalking is not warranted under Moralism, however. To see why, consider stalkers who do have experiences as though the target loves them, and either (A) cannot recognize the contrary evidence or (B) discount it. In B-type cases, the stalker *has* evidence for *p* (their experiences as of the celebrity’s affection for them) *and* against *p* (the celebrity’s actions and testimony), but irrationally ignores part of their total evidence. So, the stalker is blameworthy for that. If the stalker raises skeptical hypotheses that would undermine the celebrity’s testimony, then he is a radical skeptic in the wild. The celebrity would be justified in not considering his error possibilities seriously, as a genuine threat to her beliefs. In A-type cases, the stalker’s rationality is compromised by their delusion; one might not even be subject to epistemic blame if the delusion is strong enough. Finally, even delusional brains-in-vats are epistemically worse-off than their non-delusional counterparts. Thanks to a helpful anonymous referee for raising this issue.

11 See, e.g., Fumerton (2005), who, a kind of skeptic, defends a normative conception of epistemic justification.

12 This Moralist would be akin to Rinard’s (2021) Pragmatic Skeptic in that she accepts that our external world beliefs are unjustified, but denies that they should be given up. This view is weaker than Rinard’s, however, because it doesn’t need to embrace the view that there are only pragmatic reasons for belief (see Rinard 2015).

13 Practical incoherence can lead thinkers to attitude change. Sometimes it leads them to tolerate incoherence, utilizing active suppression. See McGrath (2017) §4–6. While the Moralist’s dilemma makes clear for anti-skeptics the ethical costs of skepticism, provides reasons for the undecided to appreciate the serious ethical costs of skepticism, it also acts as a novel opportunity for skeptics to recognize and reduce their dissonance.

14 Although Cartesian skepticism is typically presented as a thesis about external world knowledge, many epistemologists think that it can be understood as a thesis about justification (Wright 1991; Pryor 2000), or rationally grounded belief (Pritchard 2015). Here, I am bracketing the historical methodological form of Cartesian skepticism, which uses hyperbolic doubt as a means of reaching knowledge, rather than only removing belief.

15 There is disagreement among scholars of Pyrrhonian skepticism about whether we should understand equipollence as rationally demanding suspension of judgment, or as only psychologically invoking suspension of judgment. See Machuca (2019), pg. 42. Ribeiro (2021) argues that Pyrrhonian suspension might not have such wide reach because of the “hard,” doubt- resistant features of human life” (2021, 49).

16 Some philosophers argue that epistemic norms don’t guide what we are to think. See, e.g., Hughes (2022). This arguably strays from the mainstream way of thinking about epistemic normativity, on which epistemic norms are guiding.

17 To say that these convictions are “integral to who we are”, I mean that they partly constitute our narrative identities. I assume that being convinced that *p* is to believe that *p* with certainty. ‘Certainty’ here is not an epistemic notion. It does not imply that one’s evidence guarantees that *p*, or that one’s belief that *p* couldn’t be false, only that one is highly confident that *p*.

18 See: Williams (2005 [1978]) . Although some philosophers, like Moore (1939), sometime wrote as though the target of Cartesian skepticism was the *external physical world* as such. Properly understood, the target is more specifically propositions about anything whose existence does not constitutively depend upon one’s thinking about the contents of those propositions. This will surely include every physical thing but also potentially some non-physical things, like numbers, universals, or souls.

19 The BIV and Evil Demon Hypotheses are arguably vivid proxies for more abstract possibilities of systematic error or else some other kind of epistemic disconnection from reality. See, e.g., Nagel (1986).

20 This point is central to Wright’s (2004) views about epistemic entitlement. For Wright, the Cartesian skeptical argument shows only that you cannot get a warrant to believe ~ SH, but that it strictly doesn’t follow from this that you lack warrant to believe ~ SH, since the warrant might be “unearned”, in the sense that it is not the result of any cognitive achievement but something you have by default.

21 This arguably aligns with Descartes’s way of thinking about the skeptical reasoning vis-à-vis permissible attitudes. Descartes talks about “holding back assent” (*assensionem cohibendam*) and “abstaining from forming a judgment” (*de donner mon iugement*), in Adam and Tannery VII,18 and IX, 47. See Adam, Charles and Tannery, Paul (eds.) (1897–1910). *Œuvres de Descartes*, Vols. I–XII. Paris: Leopold Cerf.

22 Rinard (2021) has developed a skeptical position at odds with this picture. The Pragmatic Skeptic holds that none of our external world beliefs are justified, because justification is a function of being favored by the evidence, and yet our external world beliefs are not, according to Rinard, favored by the evidence. Call this the Evidential Thesis. Nevertheless, Rinard argues, what we ought to believe is determined entirely by pragmatic considerations. Thus, it doesn’t follow from the Evidential Thesis and skepticism that we ought not have external world beliefs. While I’m sympathetic to this position, I think it’s important to see that Rinard’s view is a response to radical skepticism, rather than a view about what radical skepticism consists in. The argument in the text is about what skepticism consists in, i.e., what kind of challenge we are presented with.

23 Although see Ribeiro (2021), who argues that the scope of Pyrrhonian suspension might be quite narrow. Moreover, he argues that Hume and Montaigne see Pyrrhonian suspension as practically limited because of features of our doxastic agency. I think he’s right about this. The Moralist is a cousin to the tradition that recognizes the limits of doxastic agency because it emphasizes the prudential limits of epistemic rationality. Moralism is however better placed in the tradition which views the purely rational agent with suspicion, akin to pragmatists like James and other kinds of critics, like Rinard (2022) and Wolf (1982).

24 See Annas & Barnes (2000).

25 I take no substantive view about what suspending judgment is here except that it entails neither believing nor disbelieving *p*. It is compatible with what I argue that it’s a metacognitive attitude about one’s lack of belief and disbelief that *p,* as Raleigh (2021) argues, or a sui generis indecision attitude towards *p*, as Friedman (2013) argues.

26 See Butler (2002); Friend (2013); and Boag (2007).

27 For this idea, see Perin (2010). Perin understands non-evident propositions as any propositions which require inference (pp. 84–85). As long as non-evident propositions are understood as possibility false and subject to evidential norms, then it won’t matter to the main argument whether we understand the Pyrrhonian as suspending judgment about whatever goes beyond appearance or to what is non-evident. Of course, some Pyrrhonists might think that there’s not enough disagreement about these matters to really initiate an equipollent appearance to balance with the appearance of other people, love, friendship and so forth. That may be. The point remains that prima facie at least, they’d need to suspend judgment about whether other people exist, and so whether they have any loved ones, friends, etc.

28 See also Bauer (2017) and Taylor (1992). However, not everyone thinks that authenticity is an ethical ideal. See Varga (2012) for the worry that authenticity is too self-centered to be an ethical ideal.

29 Thanks to an anonymous referee for raising these two worries.

30 This is widely recognized in moral philosophy, at least when understood as mutual responsibility between friends. For an explicit defense, see Seidman (2013).

31 See Keller (2004) for the idea the friendship requires certain epistemic practices.

32 Li would also be doing something wrong if he communicated his non-belief, but the key claim is that Li’s persistent lack of commitment seems like a wrong done to his friend even if it’s not communicated. Overtime, recognition and reciprocity between people who engage in actions that any reasonable person would qualify as close-friendship is something that the participants can reasonably expect of each other. Thanks to an anonymous referee for noting the difference between secret non-belief and communicated non-belief.

33 cf. Calhoun (1995) on ‘standing for’ something.

34 Heil (1983) calls ‘consequentialism’ about reasons for belief the view that what you ought to believe is a function of both practical and epistemic reasons, such that they can weigh against each other. ‘Nonconsequentialists’ say that what you ought to believe is a function of only fulfilling epistemic norms (1983, p. 754). The Abrogationist is thereby committed to the first view.

35 As many have pointed out, a lack of evidential transmission between *p* and *q*, where *p* entails *q*, doesn’t imply that *q* is not competently deducible from *p*. See, e.g., Pritchard (2015). See also Carter & Pritchard (2016).

36 Now, the encroachment *mechanism* here cannot be as narrow as *raising the epistemic standards*. Gardiner, for instance, summarizes the mechanism as follows, that if “a belief might wrong a person or group, the threshold for justified belief is higher than for a belief that is morally neutral” (Gardiner 2018, 8). But the threshold for justified belief that meets the skeptic’s standards is as high as one might ever demand. Surely *that* standard is just too high. The threshold for (II*), for example, would of course exceed ordinary standards, but so much the worse for it. We should rather think of the encroachment mechanism as sensitive to ways in which one’s doxastic economy can harm. Just as forming a belief can harm (coming to believe II) so too eliminating a belief (like eliminating II*) can harm. For this reason, the encroachment mechanism inverts in cases where the question is about whether we’re think about beliefs-formed or beliefs-kept. Forming (II) renders one subject to higher epistemic standards; eliminating (II*) renders one subject to lower standards—at least lower than the skeptic’s.

37 One might worry that the implication isn’t obvious. [P] ‘my friend Amu cares about me’ might be compatible with Berkeley’s idealism, Chalmer’s (2018) structuralism, or Walker’s (2020) disjunctionism, and so not *entail* that there’s an external physical world. The Moralist should say that we are minimally committed to the existence of whatever we quantify over with our personal beliefs.

38 Consider an analogy: suppose the only thing holding a general back from using nuclear weapons is a specific argument *A* for theory strategist military theory about why we shouldn’t use such weapons. Wouldn’t it be better to have additional more diverse arguments (ones that aren’t only about military strategy)? Sure enough, there will be disputes about which theory is best (explanatory virtues, etc.) but all-things-considered more is better. This better safeguards the relevant decisions, beliefs, and practices.

39 Some philosophers, like Rinard (2021, 2017a, 2017b) and Fumerton (2005), agree with external world skeptics to the extent that they think our external world beliefs are epistemically unjustified.

40 I ignore the complexities of these theories here because they’re not relevant to the response. The main point is that the dispute rests on subjectivism about the good life. It doesn’t follow that preference-satisfaction and pleasure don’t factor into whether one flourishes, only that it’s insufficient.

41 The ethics of radical doubt fruitfully connects with the applied dimensions skepticism in other ways, like skepticism about rape-accusations (Gardiner 2021), climate-change skepticism (Worsnip 2021), or certain metaphysical outlooks, like solipsism. Some argue that solipsism leads to radical skepticism (Helton 2021). For an exploration of some of the ethical features of solipsism, see Langton (1997). While the arguments here to don’t speak to that view, there’s a natural route from the risk of belief-revision, perhaps grounded in the prudential costs of cognitive adjustment and the harms of dehumanizing other people with lack of belief in their existence and suffering. Many thanks to an anonymous referee for raising this possibility.

42 I’d like to sincerely thank two anonymous referees of *Erkenntnis* for really their helpful suggestions along with referees at another journal, members of the Zurich Epistemology Group on Rationality (ZEGRa) and Léna Mudry especially for commentary on an earlier version of this paper. Thanks also to Nadja El-Kassar, Giada Fratantonio, Diego Machuca, Avram Hiller, and Mark Walker for their helpful comments on an earlier manuscript.
